# Psychiatric Symptoms and Cognitive Disorders in Behçet’s Disease: A Single-Center, Cross-Sectional Study

**DOI:** 10.3390/jcm12093149

**Published:** 2023-04-27

**Authors:** Fanny Urbain, Isabelle Hardy-Léger, Ghaidaa Adebs-Nasser, Mathilde de Menthon, Cécile Pivert, Aude Mausoléo, Ariane Laparra, Nathalie Lerolle, Paul-Albert Domnariu, Olivier Lambotte, Christian Denier, Cécile Goujard, Alicia Castro-Gordon, Nicolas Noel

**Affiliations:** 1 Internal Medecine and Clinical Immunology Department, AP-HP, Hôpital Bicêtre, Groupe Hospitalier Universitaire Paris Saclay, F-94275 Le Kremlin-Bicêtre, France; 2 Faculty of Medicine, Paris Saclay University, F-94275 Le Kremlin-Bicêtre, France; 3 Neuro-Imaging Department, AP-HP, Groupe Hospitalier Universitaire Paris Saclay Hôpital Bicêtre, F-94275 Le Kremlin-Bicêtre, France; 4 Immunologie des Maladies Virales et Auto-Immunes (IMVA), INSERM UMR 1184, Université Paris Saclay, F-94275 Le Kremlin-Bicêtre, France; 5 Division of Immuno-Virology, IDMIT, CEA, DSV/iMETI, Université Paris-Saclay, F-94275 Le Kremlin-Bicêtre, France; 6 Neurology Department, AP-HP, Groupe Hospitalier Universitaire Paris Saclay, Hôpital Bicêtre, F-94275 Le Kremlin-Bicêtre, France; 7 Centre d’étude en Santé des Populations (CESP), INSERM UMR 1018, Université Paris-Saclay, F-94275 Le Kremlin-Bicêtre, France

**Keywords:** Behçet’s disease, cognitive impairment, neuropsychiatry, psychopathology

## Abstract

Background: Behçet’s disease (BD) is a rare form of vasculitis involving both veins and arteries of all calibers. Psychological symptoms and cognitive impairment appear to be frequent, but few data are available. Methods: All consecutive patients in our center fulfilling the 2013 BD criteria underwent a psychometric evaluation with auto- (SCL-90-R and Modified Fatigue Index) and hetero-questionnaires (MINI). A standardized test battery assessed cognitive dysfunction. Data were correlated with BD activity as well as quality of life (SF-36). Results: We included 20 consecutive patients (16 men, four women) with a median [IQR] age of 38 (30.0–45.5) and a median disease duration of 7 years (1.8–11.0). Five patients had an abnormal brain MRI. The SCL-90-R questionnaire highlighted eight psychopathological profiles (42.1%) that correlated with altered quality of life and more severe fatigue. The most frequent symptoms were anxiety (9/19, 47.4%), somatization (8/19, 42.1%) and phobia (5/19, 26.3%). Psychopathological symptoms appeared to be more severe, but not more frequent, in neuro-Behçet’s patients. Based on standardized cognitive evaluation, nine patients had cognitive impairment defined by three or more altered tests. Notably, 6/9 patients did not have any complaint of memory loss and were thus considered ansognostic. Conclusion: Cognitive involvement was significantly associated with BD activity score (BSAS) but not with brain MRI abnormalities.

## 1. Introduction

Behçet’s disease (BD) is a rare form of vasculitis involving both veins and arteries ranging from small to large in size. The main symptoms include bipolar aphthosis, cutaneous symptoms (erythema nodosum, pseudofolliculitis, papulopustular lesions and acneiform nodules) and ocular involvement (mainly anterior and posterior uveitis) [1].

Neurological involvement occurs in 5–50% of patients and it has been included as criteria of BD diagnosis. The spectrum includes parenchymal lesions such as rhombencephalitis, pseudotumors and myelitis, while extra-parenchymal lesions typically consist of cerebral venous thrombosis and arterial aneurysm. These lesions may be associated with oligosymptomatic meningitis in about 70% of cases. The main symptoms are brainstem lesions such as sensori-motor deficiencies, cranial nerve palsies and sphincter dysfunctions; however, headache, confusion, personality changes and memory loss are also frequent symptoms [2,3].

Psychiatric symptoms and cognitive impairment appear to be frequent in BD. Their first description occurred more than 50 years ago [4]. Descriptive data are scarce, especially for people living in France [5]. Symptoms of anxiety and depression, as well as psychotic disorders, seem to be the most frequent symptoms in BD [5]. The link with the overall disease activity or the presence of parenchymal NBD lesions is unknown. Indeed, psychiatric and cognitive involvement in BD seems possible without the morphological involvement observed in MRI for other autoimmune or inflammatory disorders such as lupus [6].

We therefore designed this study in order to reveal and quantify psychiatric and cognitive disorders in our BD patient cohort.

## 2. Materials and Methods

### 2.1. Patients and Data

#### 2.1.1. Patients

Inclusion criteria: We included all patients referred to our center diagnosed with Behçet’s disease between June 2020 and December 2020. All patients fulfilled the international BD criteria [3].

Exclusion criteria: There were no exclusion criteria other than an insufficient level in French (the language in which the questionnaires were assessed) or an inability to consent to the study. Raw data from this study are available upon request to the authors.

The psychiatric and cognitive evaluations were performed during a planned annual evaluation of BD. The annual review combined physical examination for Behçet’s disease activity, blood tests, cerebral MRI and ophthalmologic evaluation. The BSAS [7] (Behçet’s Syndrome Activity Score) has a maximum score of 100 and includes 10 questions concerning the manifestation of ulcers, skin lesions, abdominal pain or diarrhea, red eyes or blurred vision and blood clots or swelling/discoloration of the extremities over the previous four weeks as well as any perceived disease activity, while the BDCAF [8] (Behçet’s Disease Current Activity Form) has a maximum score of 20 and includes questions concerning any current manifestation of headaches, ulcerations, erythema, pustulosis, arthralgia or arthritis, abdominal symptoms, new eye involvement, new neurological involvement and new major vessel involvement; these scores were used to quantify the overall BD activity that had been previously published. Notably, there is no threshold defining “active Behçet’s disease” for these two scores. Particular attention was paid to neurological evaluation.

#### 2.1.2. Psychiatric Evaluation

During their annual evaluation, all patients participated in questionnaires evaluating their fatigue (Modified Fatigue Index 20 [9]—self-assessment) and quality of life (Short Form 36 [10]—self-assessment). The 36-Item Short-Form Health Survey questionnaire evaluates nine domains: physical functioning, role limitations owing to physical health, role limitations owing to emotional problems, energy/fatigue, emotional well-being, social functioning, pain, general health and perception of health changes. Each subscale ranges from 0 (worst health state) to 100 (best health state) for each dimension. The 20-item Multidimensional Fatigue Inventory questionnaire evaluates four domains: general fatigue, reduced activity, motivation and mental fatigue. Each subscale ranges from 1 (best response to fatigue) to 5 (worst response to fatigue) for each domain [9].

We searched for symptoms of depression using part of the Mini International Neuropsychiatric Interview [11] (MINI) hetero-questionnaire. This questionnaire concerns the manifestation of depressive symptoms over the previous 15 days and any history of depression.

The Symptom Checklist-90-R (SCL-90-R) [12] questionnaire was used to investigate for the nine common dimensions of psychiatric symptoms; i.e., somatization, obsessive-compulsive behavior, social inhibition, depression, anxiety, hostility, phobic anxiety, paranoid ideation, psychoticism. It also contains three specific global indexes: the Global Severity Index (GSI) measures overall psychological distress; the Positive Symptom Distress Index (PSDI) measures intensity of symptoms; and the Positive Symptom Total (PST) measures the number of self-reported symptoms. The SCL-90-R was obtained from https://www.pearsonclinical.fr/ on 1 January 2020 with permission.

#### 2.1.3. Cognitive Evaluation

A specialized neuropsychologist (IHL) performed a standardized cognitive examination. Memory complaints were assessed by the Mac Nair questionnaire [13]. This 39-item scale is a subjective tool that measures the subject’s memory complaints in daily life. It explores various cognitive domains such as attention, focus, language, orientation in time, prospective memory and orientation concerning other people. Thereafter, neuropsychological assessment was performed via a standardized test battery [14]. These tests were chosen on the basis of their sensitivity in measuring specific cognitive domains and in their test–retest reliability. They are validated by the French group for general cognitive evaluation. The neuropsychological evaluation lasted approximately one hour per patient. The order of administration of the tests was identical for all patients in the study. The following tests, which measure learning and memory, processing speed and executive function, were selected: the Hopkins Verbal Learning Test-Revised (HVLT-R), the Trail Making Test (TMT) and the Controlled Oral Word Association (COWA) of the Multilingual Aphasia Examination. The precise aim of each test is shown in Table 1. Each test was adjusted according to age, sex and education.

Cognitive impairment was defined by three or more tests with insufficient performance (below the fifth percentile of the normal population). Notably, significant cognitive complaint was defined by a Mac Nair test > 59, according to the literature [13]. Anosognosic patients were defined by a lack of cognitive complaint but defined cognitive impairment.

#### 2.1.4. Magnetic Resonance Imaging (MRI)

All but one patient underwent a brain MRI on the day of neurological evaluation ± 1 month due to logistical constraints. The sequences were axial diffusion, FLAIR, T2*, 3D Swan T1, 3D T1 and T1 after gadolinium injection. Typical parenchymal lesions in neuro-Behçet’s are in hypoT1 and hyperT2 signals, commonly associated with vasogenic edema and typically moderate patchy enhancement. The brainstem (pons), basal ganglia and thalamus are usually involved. Other disorders of the central nervous system include central vein thrombosis and meningoencephalitis [15]. Brain atrophy was qualitatively assessed by a specialized neuroradiologist (GA-N).

### 2.2. Statistical Analysis

As the study was proposed to every patient in our cohort, no sample size was calculated. Quantitative and qualitative data were presented as median [interquartile range, IQR] or in percent (%). The between-groups comparisons were performed using the Mann–Whitney U, the χ² or exact Fisher test when appropriate. Correlations were made using Spearman coefficient analysis. For all results, statistical significance was set to *p* < 0.05. All the data analyses were performed with Graphpad (version 9.0, LaJolla, CA, USA).

### 2.3. Ethics

This study received approval from from the ethics committee (N° ID-CRB: 2019A01819-48) according to the French Jardet law. All patients gave written consent for their participation.

## 3. Results

### 3.1. Patients’ Description

We included 20 patients (16 men/four women). The median [IQR] age was 38 (30–45.5). The median disease duration from BD diagnosis was 7 (1.75–11) years. None of the patients refused the study. Their clinical, biological and imaging data are summarized in Table 2.

At BD diagnosis, all but one patient had oral aphtosis. Twelve (60%) had genital aphtosis, and 10 (50%) had ocular involvement. Seven (35%) had skin involvement, and eight (40%) had vascular involvement. Four patients (20%) had inaugural neurological involvement; three had parenchymal infarction, and one had extra-parenchymal infarction. Other involvement includes arthralgia (n = 2, 10%), fever (n = 1) and seritis (n = 1).

Regarding the treatment, 17/20 patients (85%) received colchicine, 14/20 (70%) had received steroids (nine still ongoing), and 13/20 (65%) had other immunosuppressive treatments, mainly infliximab (n= 7, 35%), azathioprine (n = 7, 35%), methotrexate (n = 5, 25%), cyclophosphamide (n = 3, 15%) and adalimumab (n = 2, 10%). One patient had consecutively received infliximab and adalimumab. Indication and choosing of treatment relied on international and national recommendations [16,17]. Three patients (15%) had stopped immunosuppressive treatment at the time of examination since their disease had improved. Half the patients had received one therapeutic line (n = 7, 35%), but some needed changes in their treatment, with two lines (n = 3, 15%), three lines (n = 2, 10%) and four lines (n = 1, 5%) of therapy.

Thirteen (65%) were currently smoking and four (20%) engaged in daily alcohol consumption; among them, three had stopped. Three patients (15%) engaged in daily consumption of cannabis. Lastly, only two patients (10%) received psychotropics at the time of the study: one patient was treated with alprazolam and the other one with bromazepam, tropatepim, risperidone, duloxetine and loxapine. Both were already followed for anxiety and depression with psychoticism, respectively.

At study enrollment, symptoms reported were: active mouth ulcers n = 10 (50%), genital ulcers n = 2 (10%), folliculitis n = 6 (30%), headache n = 7 (35%) and arthralgia n = 5(25%). No patient had active uveitis.

The overall BD activity was moderate; the median BSAS was 12.75 (4.75–36) and the median BDCAF was 5 (3–7.5).

Five patients had abnormal MRI consisting of: cerebral thrombophlebitis sequelae with left occipital and cerebrum infarction (n = 1), left frontal and brainstem FLAIR hypersignal (n = 1), mesencephalic FLAIR hyper signal (n = 1) and right temporo-parietal microbleed (n = 1). For one patient, the neurological involvement was found during this systematic evaluation, without clinical symptoms, with antero-inferior pons FLAIR hypersignal. One patient had cerebral atrophy. These five patients were considered to have cerebral involvement and were therefore classified as the neuro-Behçet’s (NBD) group.

**Figure 1:**
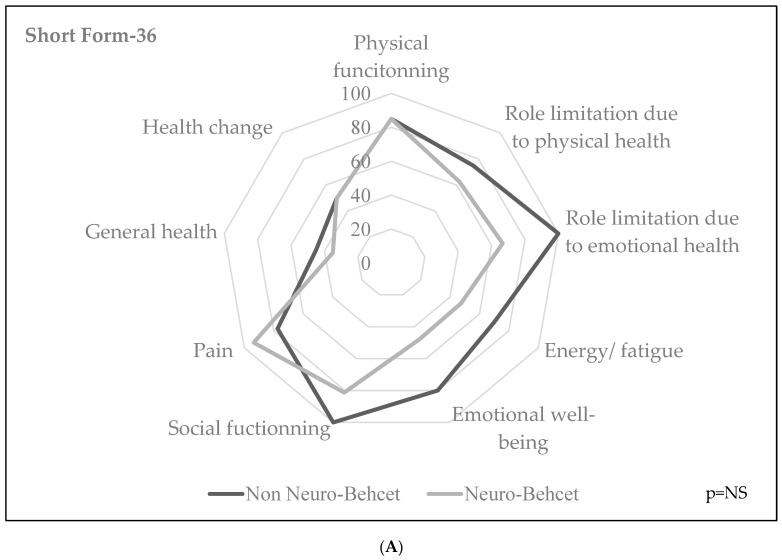

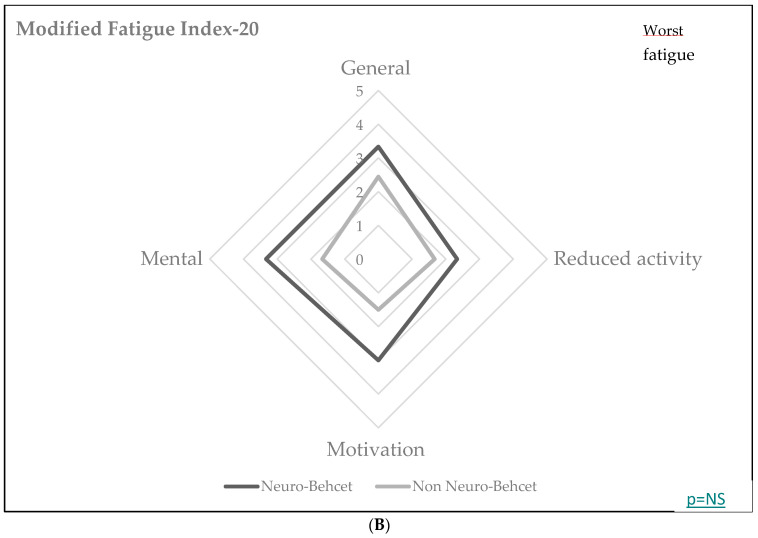
Quality of Life (SF-36) (**A**) and Fatigue (MFI-20) (**B**) with or without neuro-Behçet’s. Short-form 36 (SF-36) is an auto-questionnaire about health-related symptoms. Each subscale ranges from 0 to 100, corresponding to the worst and best health state in each dimension, respectively. The 20-item Multidimensional Fatigue Inventory (MFI-20) questionnaire evaluates four domains: general fatigue, reduced activity, motivation and mental fatigue.

The graphical representation for quality of life and fatigue dimensional analyses are represented in Figure 1A,B. Patients with NBD seemed to display more fatigue in their MFI scores, especially concerning general and mental fatigue, without statistical significance.

### 3.2. Psychiatric Symptoms

According to the MINI score, one patient (5%) had symptoms of severe depression in the previous 15 days. This patient was already followed up and treated for depression and psychotism and had a normal brain MRI.

**Figure 2:**
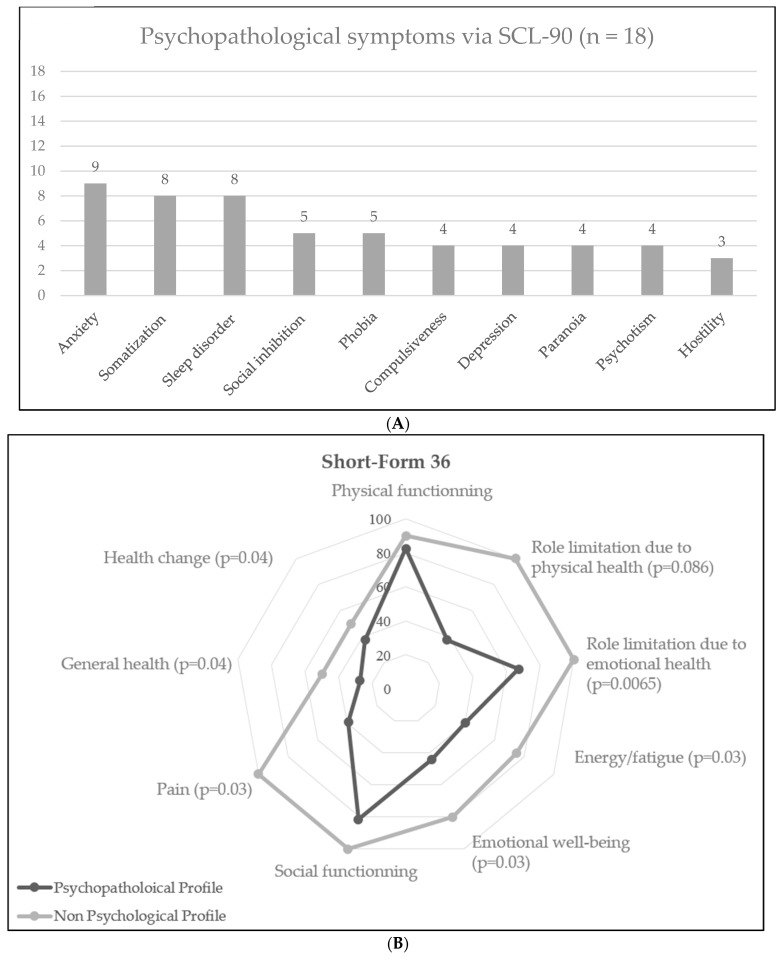

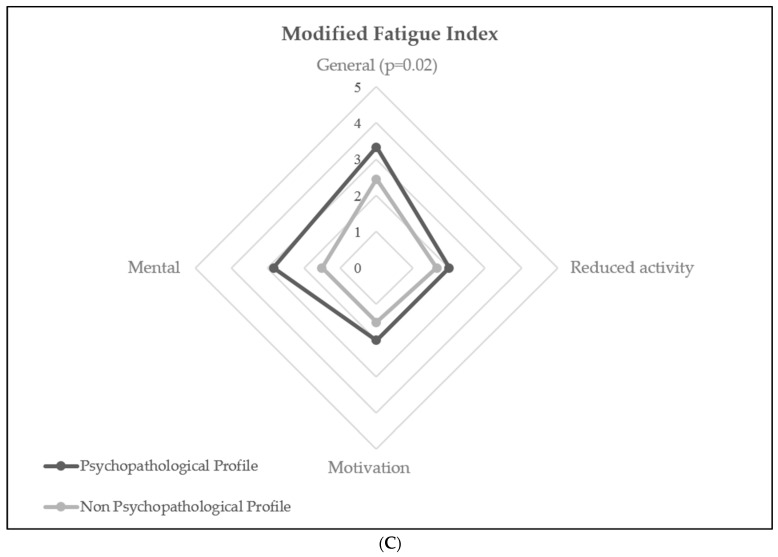
Psychopathological profiles of the cohort (SCL-90-R). (**A**): Frequency of psychopathological symptoms amongst patients according to SCL-90-R test (n = 18). (**B**): Comparison in health-related symptoms via Short-Form 36 between patients with and without psychopathological profiles. (**C**): Comparison in fatigue symptoms via Modified Fatigue Index between patients with and without psychopathological profiles.

The nine-dimension analysis of psychiatric symptoms through the SCL-90-R questionnaire found that the main psychopathological symptoms were anxiety (nine patients, 47.4%), somatization (eight patients, 42.1%) and phobia (five patients, 26.3%). The nine dimensions are depicted in Figure 2A.

Overall, eight patients (42.1%) were considered to have significant psychopathological symptoms. The median Global Severity Index (GSI) was 0.41 [0.20–0.70]. Severity was pathological for two (10.5%), moderate for seven (36.8%) and absent for nine (47.4%). Intensity was pathological for four (21.1%), moderate for eight (42.1%) and absent for six (31.6%). Eight patients (42.1%) had sleep disorders.

We then analyzed the factors associated with psychopathological symptoms. As shown in Table 2, there was no difference in terms of psychiatric symptoms between NBD and non-NBD patients. However, patients with NBD had a higher global severity index in their psychopathological scores (0.67 [0.63–0.70] vs. 0.26 [0.16–0.60], *p* = 0.03), with more frequent pathological/moderate severity than non-NBD patients in their psychiatric symptoms. The psychopathological profile of BD was not related with sex, age at study enrollment or disease duration, but there was a positive correlation with disease activity (median [IQR] BSAS score: 26.5 [4.3–45] vs. 10.8 [2.3–19.8], *p* = 0.18—Supplementary Table S1). There was no relation with past or present medication either.

However, as shown in Figure 2B,C, the psychopathological profile was associated with significant alteration in quality of life (mostly feeling of role limitation due to physical health and emotional health, emotional well-being, pain, feeling of altered general health and health changes) and fatigue (reported in both the SF-36 and the MF-I20 questionnaires, mostly due to general and mental fatigue).

### 3.3. Cognitive Evaluation

Lastly, we analyzed whether BD patients had cognitive complaints and/or troubles. One patient could not undergo cognitive evaluation due to severe cognitive impairment (unable to understand tests) and illiteracy.

According to the Mac Nair test, 4/19 (21.1%) patients had memory complaints. None of them had neuro-Behçet’s. The memory span (evaluating short-term and working verbal memory) was below average or insufficient for eight patients. The Weschler Adult Intelligence Scale (WAIS) subtest (Code test—exploring learning and strategy and information processing speed) was insufficient for 7/18 patients (38.9%). Phonemic and semantic speeded lexical fluences were insufficient for three (15.8%) and eight (42.1%) patients, respectively. Twelve patients (63.2%) failed in the Hopkins Verbal Learning Test, which measures learning and memory. Concerning the Trail Making Test (TMT), which measures psychomotor speed and aspects of executive function, seven patients (41.2%) failed. No difference was observed whether the patient had NBD or not (Table 3). The results were not influenced by cognitive complaints (Supplementary Table S2).

Finally, 9/19 (47.3%) had insufficient performance for three or more tests, fulfilling criteria for cognitive impairment. Factors associated with cognitive impairment are summarized in Table 4. Once again, the presence of brain lesions on MRI (NBD) was not associated with cognitive troubles. There was no difference with respect to sex ratio, age or cognitive complaint. Interestingly, however, the global activity of BD using the BSAS score was significantly associated with altered cognition (median BSAS score: 31 [12.8–42] and 7 [1.9–21.8], respectively; *p* = 0.046) (Figure 2). Notably, there is no validated definition of an “active disease” in the literature. As for psychopathological symptoms, we did not find any association between cognitive impairment and corticosteroid intake or immunosuppressive lines (Table 4).

Importantly, 6/9 patients (66.7%) who had three or more insufficient performances in objective cognitive tests had no complaint in the Mac Nair questionnaire and were therefore considered anosognostic. Among them, only one patient had abnormal MRI. They did not have any specific profile in terms of MFI-20 and QoL assessments. According to SCL-90-R, three had symptoms of anxiety and two had a psychopathological profile.

## 4. Discussion

Here, we report the first systematic psychiatric and cognitive evaluation for patients with BD in France. Our cohort was representative of the average rate of neurological involvement in BD, present in 25% of our patients [5].

Psychopathological symptoms appeared to be frequent in Behçet’s disease. In our cohort, the main symptoms reported were anxiety, somatization and phobia. Moreover, 42.1% of patients presented with a significant psychopathological profile according to the SCL-90-R assessment. In comparison, Dursun et al. [18] reported that 30/76 patients from a Turkish cohort presented with at least one psychiatric disorder (based on the DSM-IV), mostly major depression (17.8%), specific phobia (16.4%) and generalized anxiety disorder (15.1%). Most studies examined differences between frequency of depression and anxiety between BD patients and healthy controls [19,20,21] and found a significant increase in depression, anxiety and fatigue rates in BD patients. Comparisons with other inflammatory diseases have been performed to highlight differences between pathophysiological pathways. In two studies comparing BD to psoriasis patients [22,23], depression and anxiety scores were significantly higher in the BD group. Comparison with systemic lupus erythematosus (SLE) shows inconsistent results, with some studies suggesting a higher proportion or depressed/anxious patients in SLE [24] and one revealing a higher proportion of bipolar patients in BD than in SLE [25]. In our cohort, there was no correlation between Behçet’s neurological involvement and presence of psychopathological profile, even though they appear to have more severe symptoms. We did not find relation with sex, age or duration of disease. More severe activity tends to be associated with presence of psychopathological symptoms, without reaching significance. Correlation with activity is inconstant in the literature [18,24,25]. The association between severity/intensity of psychiatric symptoms and quality of life emphasizes the impact of such symptoms in daily living. Physicians should systematically search for psychopathological symptoms in order to propose proper care and support.

Neurocognitive impairment was frequent in our cohort, affecting nearly 50% of patients, despite a severe definition of cognitive impairment. The most altered functions were learning and memory (n = 12, 63.2%), attention (n = 7/17, 41.2%) and semantic fluencies (n = 8, 42.1%). Interestingly, this was not associated with neurological involvement but with overall disease activity. Published data about cognitive impairment in BD are consistent with our findings. Monastero and al. [6] found that 12/26 (46.1%) BD patients with normal brain MRI had cognitive impairment, defined by two or more pathological tests among a standardized battery. Cognitive impairment was associated with high disease activity and high prednisone dosage. Cavaco and al. [26] discussed a protective role of current prednisone intake for cognitive impairment but still noticed the high prevalence of cognitive dysfunction in BD, either with or without neurological involvement (8/15 and 14/35, respectively). Other studies confirmed a range of cognitive disorders between 40 and 50%, irrespective of the neurological involvement [20,21,27]. In comparison with multiple sclerosis, Gündüz et al. [28] found a more dysexecutive/frontal syndrome in Behçet’s patients. In our study, we did not find any association between cognitive impairment and corticosteroid intake or immunosuppressive lines. The link we observed with disease activity suggests a potential role for systemic low-grade inflammation in cognitive dysfunction in BD.

Another important finding in our study is the absence of correlation between cognitive impairment and memory complaints since 6/9 patients with cognitive impairment were anosognostic. This highlights the major role of physicians in systematic evaluation of cognitive impairment. These data advocate for reinforcement in the screening of cognitive impairment in BD patients irrespective of the presence of neurological lesions or memory complaints.

Our study has several limitations. Our cohort is small, and our findings should be confirmed by larger studies. Systemic or CNS inflammation markers (such as intrathecal cytokines, markers of neuronal dysfunction and cytology) and dynamic imaging (TEP-MRI) were not available in this study. Further studies are needed to explore relationships between inflammation and cognition dysfunction in BD. We also lack comparability with other previously published cohorts as there is no generalized standardized neurocognitive battery published for BD, in contrast with SLE, for example [29]. Finally, our study is cross-sectional, and no follow-up is currently available for patients without cognitive involvement to detect any appearance of cognitive decline in the future. Nevertheless, the strength of this study is in the use of systematic psychometric and cognitive evaluation in patients with or without morphological alterations of neuro-BD and in highlighting the high proportion of anosognosic patients.

## 5. Conclusions

Patients with Behçet’s disease frequently present with psychological disorders and cognitive impairment, irrespective of neurological involvement. The presence of psychopathological symptoms was associated with altered quality of life. Cognitive complaints were rare and not associated with cognitive dysfunction, suggesting that some patients would not be detected in the absence of such systematic evaluation. Physicians should carry out systematic investigations in order to propose proper care. These results should be validated in a larger cohort in order to ascertain cognitive involvement and highlight predictive factors.

## Figures and Tables

**Table 1:** Summary of all neurocognitive test batteries.

Mac Nair Test	Cognitive and Memory Complaint
Memory span (numbers)	Short term and working memory
Hopkins Verbal Learning Test	Learning and memory test - Retrieval trouble (recognition is normal) - Learning trouble (recognition is abnormal)
Trail Making Test	Simple and alternate attention test
Phonemic and semantic fluencies	Information retrieval organization and strategy
Weschler Adult Intelligence Scale (Code test)	Learning and strategy test. Information processing speed test
Psychiatric evaluation	
Modified Fatigue Index (MFI)—20 items	Auto-questionnaire about fatigue
Mini International Neuropsychiatric Interview (MINI)	Hetero-questionnaire about symptoms of depression
Symptom Checklist-90-R (SCL-90-R) questionnaire	Auto-questionnaire about psychiatric symptoms
General evaluation	
Short-form 36 (SF-36)	Auto-questionnaire about health-related quality of life
Behçet’s Syndrome Activity Score (BSAS)	Activity of Behçet’s disease
Behçet’s Disease Current Activity Form (BDCAF)	Activity of Behçet’s disease

**Table 2:** Patient Characteristics.

	Patients n = 20
Demographical data	
Sex (men/women)	16/4
Age (years) median [IQR]	38 [30–45.5]
Disease duration (years) median [IQR]	7 [1.75–11]
Habitus (past or present)	
Tobacco use n (%)	13 (65%)
Alcohol consumption n (%)	4 (20%)
Cannabis use n (%)	4 (20%)
Treatments	
Colchicine n (%)	17 (85%)
Current steroid use n (%)	9 (45%)
Current immunosuppressive treatment	10 (50%)
Infliximab (present or past) and/or adalimumab n (%)	8 (40%)
Lines of immunosuppressive treatment Psychotropic treatment n(%)	1 line (n = 7, 35%), 2 lines (n = 3, 15%), 3 lines (n = 2, 10%) and 4 lines (n = 1, 5%). 2 (10%)
Active symptoms during day evaluation	
Mouth ulcers n (%)	10 (50%)
Genital ulcers n (%)	2 (10%)
Folliculitis n (%)	6 (30%)
Erythema Nodosum n (%)	0
Headache n (%)	7 (35%)
Uveitis n (%)	0
Arthralgia n (%)	5 (25%)
Evaluation	
Abnormal cerebral MRI n (%)	5/20 (25%)
CRP > 5 mg/L n (%)	3/20 (15%)
HLA B51 n (%)	3/15 (33%)
Disease Activity	
BSAS median [IQR]	12.75 [4.75–36]
BDCAF median [IQR]	5 [3–7.5]

IQR: inter-quartile range; BSAS: Behçet’s Syndrome Activity Score; BDCAF: Behçet’s Disease Current Activity Form.

**Table 3:** Psychiatric symptoms and cognitive evaluation score, depending on the presence of NBD.

	All Patients	Neuro-Behçet’s	Non-Neuro-Behçet’s	*p*
**MINI**	n = 20	n = 5	n = 15	
Present depression	1	0	1	1
Past depression	1	0	1	1
**SCL-90 n, %**	n = 18	n = 4	n = 14	
Somatization	8, 44.4%	2 (50)	6 (43)	1
Compulsiveness	4, 22.2%	1 (25)	3 (21)	1
Social inhibition	5, 27.8%	2 (50)	3 (21)	0.53
Depression	4, 22.2%	1 (25)	3 (21)	1
Anxiety	9, 50.0%	4 (100)	5 (36)	0.08
Hostility	3, 16.7%	1 (25)	2 (14)	1
Phobia	5, 27.8%	2 (50)	3 (21)	0.53
Paranoia	4, 22.2%	1	3	
Psychotism	4, 22.2%	0 (0)	4 (29)	0.52
Sleep disorder	8, 44.4%	3 (75)	5 (36)	0.27
Psychopathology	8, 44.4%	3 (75)	5 (36)	0.27
Global severity Index, median [IQR] Severity	0.405 [0.2–0.7]	0.67 [0.63–0.70]	0.26 [0.16–0.60]	**0.03**
*Pathological*	2, 11.1%	0 (0)	2 (14)	
*Moderate*	7, 38.9%	4 (100)	3 (21)	**0.02**
*Absent*	9, 50.0%	0 (0)	9 (64)	
Positive Symptom Distress Index [IQR]				
*Pathological*	4, 22.2%	2 (50)	2 (14)	
*Moderate*	8, 44.4%	2 (50)	6 (43)	0.17
*Absent*	6, 33.3%	0 (0)	6 (43)	
**Mac Nair significant complaint (>54) n (%)**	4 (21.1%)	0 (0)	4 (27)	0.53
**Memory Span n (%)**				
Satisfying	2 (10.5%)	1	1	0.55
Intermediate	9 (47.4%)	2	7	
Below average	4 (21.1%)	0	4	
Insufficient	4 (21.1%)	1	3	
**Weschler Adult Intelligence Scale subtest (Code test) n (%)**		n = 3		
Satisfying	1/18 (5.6%)	0	1	0.86
Intermediate	10/18 (55.6%)	2	8	
Insufficient	7/18 (38.9%)	1	6	
**Phonemic fluency insufficiency, n (%)**	3 (15.8%)	0 (0)	3 (20)	1
**Semantic fluency insufficiency, n (%)**	8 (42.1%)	1 (25)	7 (47)	0.60
**Hopkins Verbal Learning Test, insufficiency, n (%)**	12 (63.2%)	2 (50)	10 (67)	0.60
**Trail Making Test insufficiency, n (%)**	7/17 (41,2%)	0/3 (0)	7/14 (50)	0.23
**Insufficiency ≥ 3 tests**	9/19 (47.3%)	1 (25)	8 (57)	0.58

The between-groups comparisons were performed using the Mann–Whitney U, the χ² or exact Fisher test when appropriate. Statistical significance was *p* < 0.05. Significant comparisons are in bold. There was no difference in frequency of psychological symptoms between BD patients and NBD patients except for a trend in anxiety (*p* = 0.08). GSI was higher in NBD patients than in BD patients (*p* = 0.03). Cognitive impairment was frequent regardless of specific cerebral involvement.

**Table 4:** Factors associated with cognitive troubles.

	Cognitive Trouble n = 9	No Cognitive Trouble n = 10	*p*
Man, n (%)	8 (88.9%)	7 (70%)	0.58
Age at study enrollment, years, median [IQR]	38 [30–42]	40 [28.5–55.3]	0.65
Duration of disease, years, median [IQR]	7 [1–8]	8.5 [3.75–32]	0.16
Past use of GC n (%)	1 (11.1)	4 (40)	0.28
Present use of GC n (%)	5 (55.6)	2 (20)	0.11
Past use of IS n (%)	1 (11.1)	2 (20)	0.60
Present use of IS n (%)	4 (44.4)	4 (40)	0.84
N° therapeutic lines, median [IQR]	1 [0–2]	1 [0–1.5]	0.93
Cognitive complaint n (%)	3 (33.3)	1 (10%)	0.30
Abnormal MRI/NBD n (%)	1 (11.1%)	3 (30%)	0.58
BSAS median [IQR]	31 [12.8–42]	7 [1.9–21.8]	0.046

BSAS: Behçet’s Syndrome Activity Score; GC: glucocorticosteroids; IQR: interquartile range; IS: immunosuppressants; MRI: molecular resonance imaging; NBD: neuro-Behçet’s disease.

## Data Availability

Research data are available upon request from the corresponding author.

## Supplementary Materials

The following supporting information can be downloaded at: https://www.mdpi.com/article/10.3390/jcm12093149/s1, Table S1: factors associated with the psychopathological profile; Table S2: results from the neurocognitive evaluation, depending on the presence of cognitive complaints.
