# Supplementary material for: Psychiatric Symptoms and Cognitive Disorders in Behçet’s Disease: A Single-Center, Cross-Sectional Study
Source: J Clin Med. 2023 Apr 27;12(9):3149. doi: 10.3390/jcm12093149 (PMC10179639; doi:10.3390/jcm12093149)
Supplement: Supplementary file 1 [file jcm-12-03149-s001.zip › jcm-2289309-supplementary.pdf]

SUPPLEMENTARY MATERIAL

Supplementary table 1 : Factors associated with the psychopathological profile			
	**Psychopathological BD n=8**	**Non psychopathological BD n=12**	**p**
Man, **n (%)**	8 (100)	6 (60)	0.09
Age at study enrollment, years, median [IQR]	31.5 [30-42.5]	39.5 [30-40.3]	0.25
Duration of disease, years, median [IQR]	6 [1-7.8]	7.5 [2.5-32]	0.39
Past use of GC **n (%)**	3 (37.5)	2 (20)	0.61
Present use of GC **n (%)**	3 (37.5)	6 (54.6)	0.65
Past use of IS **n (%)**	2 (25)	1 (9.1)	0.55
Present use of IS **n (%)**	3 (37.5)	7 (63.6)	0.37
N° therapeutic lines	1 [0-1.75]	1 [0-2]	0.83
Cognitive complaint **n (%)**	3 (33.3)	1 (10)	0.30
Abnormal MRI/NBD **n (%)**	2 (25)	1 (10)	0.56
BSAS median [IQR]	26.5 [4.3-45]	10.8 [2.3-19.8]	0.18

BSAS: Behçet`s Syndrome Activity Score, GC: glucocorticosteroids, IQR: interquartile range, IS: immunosuppressants, MRI: molecular resonance imaging, NBD: neuro-Behçet's diseases

Supplementary table 2 : results from the neurocognitive evaluation depending on the presence of cognitive complaint			
	Complaint (n=4)	No complaint (n=15)	P
**Memory Span n (%)**			
Satisfiying	1	1	0.55
Intermediate	2	7	
Below average	0	4	
Insufficient	1	3	
**Weschler Adult Intelligence Scale subtest (Code test) n (%)**			
Satisfiying	1	0	0.10
Intermediate	1	9	
Insufficient	2	5	
**Phonemic fluence insufficiency, n (%)**	1 (25)	2 (13)	0.53
**Semantic fluence insufficiency, n (%)**	3 (75)	5 (33)	0.26
**Hopkins Verbal Learning Test, insufficiency, n (%)**	3 (75)	9 (60)	1
**Trail Making Test insufficiency, n (%)**	3/ 4 (75)	4/13 (31)	0.08
**Insufficiency ≥ 3 tests, n (%)**	3 (75)	6 (40)	0.30
